# Rectal prolapse treatment in elderly patients

**DOI:** 10.1186/1471-2318-11-S1-A50

**Published:** 2011-08-24

**Authors:** A Racalbuto, I Aliotta, R Lanteri, SA Carnazzo, V Minutolo, A Licata

**Affiliations:** 1 Department of Surgical Sciences, Organ Transplantations and Advanced Technologies University of Catania, Italy

## Background

Rectal prolapse in elderly patients can cause considerable discomfort causing bleeding, itching, wet anus and tenesm. In older patients the gold standard treatment uses a perineal approach. Success obtained by using circular staplers in the treatment of internal prolapses, associated or not with haemorroids or obstructed defecation, may represent a new method of choice if applied with the appropriate modifications, to the external rectal prolapse, for its speed, simplicity and possibility to be performed under local anesthesia, and even in elderly patients with debilitated conditions.

## Patients and methods

We have included in our study 11 patients older than 70 years (3 female and 8 male, mean age 76 years – range 71-84) who underwent observation for complete, permanent or after effort, external rectal prolapse. All patients suffered from rectal bleeding, in two cases considerable and with concomitant severe anemia (Hb <6.0), as well as itching and wet anus. Fecal incontinence was not present in any of the cases, but about half the patients (5 / 11) complained of "soiling". Three patients had heart disease, three bronchitis, one diabetes and one Alzheimer’s. Before surgical treatment all patients were submitted to a pelvic examination, proctological anoscopy, anorectal manometry and defecography. All patients were included in a follow up program with a clinical examination at 1 month, 6 months, 1 year and a manometry at 6 months and 1 year.

## Results

Surgical time (medium time 40 min- range 30-65 min) was lower than that of the traditional method (medium time 45 min – range 30-75 min) P= NS. Spinal anesthesia technique efficiently ensured the best conditions for the intervention. In the immediate postoperative period we observed one case of urine retention while an analgesic was necessary in three cases. 7 patients were discharged within 48 h after surgery, while four had a longer hospitalization due to poor general conditions (1) or rectal bleeding (3). At follow up (mean 26 months- range 6-32) 10 patients could be considered a complete success. One patient (sex M- age 82 years) presented recurrence at 6 months. A new treatment using the same procedure has solved the disease and he is well with no recurrences 18 months after the second procedure. Checks performed showed no difference before surgery for basal sphincter pressure (mean 63 +/- 6 mmHg preoperatively, 58+/-3 mmHg after, P=NS) and for voluntary contraction pressure (mean 127+/- 36 before and 118+/- 25 after surgery, P=NS).

## Conclusions

Our results demonstrated the advantage of our technique in elderly patients.

## References

[B1] Kimmins M Evetts BK Isler J Billingham R The Altmeier Repair: Outpatient Treatment of Rectal Prolapse Dis Colon Rectum 2001 44 565 70 10.1007/BF02234330 11330584

[B2] Racalbuto A Gresta S Papillo B Scilletta B Di Cataldo A Licata A Posterior rectopexy with Gore-Tex prosthesis for the treatment of complete rectal prolapse It J Coloproc 1997 16 115 120

[B3] Ramanujam PS Venkatesh KT Fiez MJ Perineal excision of rectal procidentia in elderly high risk patients Dis Colon Rectum 1994 37 1027 30 10.1007/BF02049318 7924710

[B4] Johansen OB Wexner SD Daniel N Nogueras JJ Jagelman DG Perineal rectosigmoidectomy in the elderly Dis Colon Rectum 1993 36 767 72 10.1007/BF02048369 8348868

[B5] Oliver GC Vachon D Eisenstat TE Rubin RJ Salvati EP Delorme procedure for complete rectal prolapse in severely debilitated patients. An analysis of 41 cases Dis Colon Rectum 1994 37 461 7 10.1007/BF02076192 8181408

[B6] Ramanujam PS Venkatesh KS Perineal excision of rectal prolapse with posterior levator ani repair in elderly high risk patients Dis Colon Rectum 1988 31 704 6 10.1007/BF02552589 3168681

[B7] Boccasanta P Venturi M Barbieri S Roviaro G Impact of new technologies on the clinical and functional outcome pf Altmeier's procedure: a randomized, controlled trial Dis Colon Rectum 2006 49 652 60 10.1007/s10350-006-0505-6 16575620

[B8] Scherer R Marti L Hetzer F Perineal Stapled Prolapse Resection: a new procedure for External Rectal Prolapse Dis Colon Rectum 2008 51 1727 30 10.1007/s10350-008-9423-0 18626711

